# Quality of life after stay in surgical intensive care unit

**DOI:** 10.1186/1471-2253-7-8

**Published:** 2007-07-24

**Authors:** Fernando J Abelha, Cristina C Santos, Paula C Maia, Maria A Castro, Henrique Barros

**Affiliations:** 1 Department of Anesthesia and Intensive Care, Hospital de São João, Porto, Portugal; 2 Biostatistics and Medical Informatics Department, University of Porto Medical School, Porto, Portugal; 3 Department of Hygiene and Epidemiology, University of Porto Medical School, Porto, Portugal

## Abstract

**Background:**

In addition to mortality, Health Related Quality of Life (HRQOL) has increasingly been claimed as an important outcome variable. The aim of this study was to assess HRQOL and independence in activities of daily living (ADL) six months after discharge from an Intensive Care Unit (ICU), and to study its determinants.

**Methods:**

All post-operative adult patients admitted to a surgical ICU between October 2004 and July 2005, were eligible for the study. The following variables were recorded on admission: age, gender, American Society of Anesthesiologists physical status (ASA-PS), type and magnitude of surgical procedure, ICU and hospital length of stay (LOS), mortality and Simplified Acute Physiology Score II (SAPS II). Six months after discharge, a Short Form-36 questionnaire (SF-36) and a questionnaire to assess dependency in ADL were sent to all survivors. Descriptive statistics was used to summarize data. Patient groups were compared using non-parametric tests. A logistic regression analysis was performed to identify covariate effects of each variable on dependency in personal and instrumental ADL, and for the change-in-health question of SF-36.

**Results:**

Out of 333 hospital survivors, 226 completed the questionnaires. Fifty-nine percent reported that their general level of health was better on the day they answered the questionnaire than 12 months earlier. Patients with greater co-morbidities (ASA-PS III/IV), had lower SF-36 scores in all domains and were more frequently dependent in instrumental and personal ADL. Logistic regression showed that SAPS II was associated with changes in general level of health (OR 1.06, 95%CI, 1.01 – 1.11, p = 0,016). Six months after ICU discharge, 60% and 34% of patients, respectively, were dependent in at least one activity in instrumental ADL (ADLI) and personal ADL (ADLP). ASA-PS (OR 3.00, 95%CI 1.31 – 6.87, p = 0.009) and age (OR 2.36, 95%CI, 1.04 – 5.34, p = 0.04) were associated with dependency in ADLI. For ADLP, only ASA-PS (OR 4.58, 95%CI, 1.68–12.46, p = 0.003) was associated with higher dependency.

**Conclusion:**

ASA-PS, age, type of surgery, ICU LOS and SAPS II could be seen as determinants of HRQOL.

## Background

Knowledge regarding the quality of life of patients treated in an Intensive Care Unit (ICU) is fundamental for judging the benefits and human costs of intensive care. Assessing patient's quality of life is a complex and often difficult task because the process involves health status and associated variables such as social and familiar relationships, employment and financial status. Health-related quality of life (HRQOL) is now recognized as an important component of outcome evaluation among survivors and can improve the assessment of quality of life [1]. Some authors state that outcome after ICU stay must include HRQOL measurements [2].

It is important to understand HRQOL in terms of specific ICU populations in order to assess the impact of specific interventions on these patients [3]. Examining non-fatal post-hospital outcomes may enable us to understand the needs and problems of ICU survivors. In recent decades, quality of life outcomes have became an issue of increasing interest because they are relevant to a better knowledge of healthcare expenditure and resource utilization. Post-operative patients are of particular interest owing to the individual risk imposed by the surgical procedure. Also, this subset of patients may differ in important ways from the general ICU population, so a study of them may give a more accurate picture of outcome and quality of life.

A large body of literature has been published in the last few years about quality of life assessment [4,5]. The study of quality of life may be generic and involving all aspects of HRQOL for a particular disease or group of patients, and several questionnaires have been validated for intensive care patients [6-12]. Most of the measures that have been used for critical care are multi-item scales; that is, they are made up of several questions or items. Some multiple-item scales provide a total score as well as generating subscales that provide information on particular aspects such as mobility. The Short-Form General Health Survey (SF-36) was developed during the Medical Outcomes Study (MOS) to measure generic health concepts relevant across age, disease and treatment groups [13]. It is a self-completed questionnaire covering all aspects of HRQOL [6,13,14]. It is a valid instrument for measuring HRQOL. It has been used for post-discharge ICU patients and groups with other diseases, shows good reliability and validity [13,15], and is recommended for assessing outcome after critical illness [16-19]. This questionnaire was culturally adapted to Portuguese and validated in a study by Ferreira [20].

The ability to care for oneself and live independently has been considered a measure of functional outcome after hospitalization and discharge from intensive care [21]. Functional status refers to the level of involvement in activities and is often used as a synonym for performance in Activities of Daily Living (ADL) [22]. ADL appraisal scales consider functional and instrumental activities. A patient' ability to handle these activities has been assessed by generic or disease-specific measures of physical functional status. Katz's Activities of Daily Living Scale [23], the Karnofsky Index [24] and Hulter-Asberg' Instrumental Index of Independence in ADL [25] have been investigated in critical care survivors.

The aim of the present study was to assess HRQOL and independence in activities of daily living among patients undergoing scheduled or emergency non-cardiac surgery, six months after discharge from the ICU, and to study its determinants.

## Methods

The protocol was approved by our institutional review board, and written consent was obtained from the patients. Post-operative patients aged 18 years or more who underwent scheduled or emergency non-cardiac surgery between October 2004 and July 2005 were eligible. Patients readmitted during the study period were enrolled in relation to the time of their first admission.

The following clinical variables were recorded on admission to the ICU: age, sex, body weight and height, American Society of Anesthesiologists physical status (ASA-PS) ([26], emergency or scheduled surgery, and magnitude of surgical procedure as categorized by Kongsayreepong et al. [27] – i.e. major (surgery in which body cavities or major vessels are exposed to ambient temperature, e.g. major abdominal, thoracic, major vascular, thoracic spine surgery with instrumentation, or hip arthroplasty), medium (surgery in which body cavities are exposed to a lesser degree, e.g. appendectomy), and minor (superficial surgery).

For all patients, we also recorded the ICU and hospital LOS and the mortality. A severity of disease score system, the Simplified Acute Physiology Score II (SAPS II), was calculated using standard methods [28].

To minimize distress to the next of kin, each patient's records were checked on the hospital information system after 6 months to ascertain whether he or she was still alive. A copy of a formal letter was sent to all known survivors accompanied by a return envelope and a validated Portuguese SF-36 self-report form [20,29]. The questionnaire also assessed the patient' employment and marital status and dependence in ADL tasks. For each patient who did not return the questionnaire and/or for whom telephone contact could not be established, the family doctor and/or the patient's relatives were contacted either to provide the correct address of the patient or to confirm death.

### Medical Outcome Survey Short-Form 36 (SF-36)

The survey contains 36 questions that evaluate eight health domains considered to be important for patient well-being and health status. These domains reflect physical health, mental health, and the impact of health on daily functioning. The eight multiple-item domains encompass physical functioning (ten items), social functioning (two items), role limitations caused by physical problems (four items), role limitations caused by emotional problems (three items), mental health (five items), energy and vitality (four items), pain (two items) and general perception of health (five items). There is one further unscaled item about self reported changes in the respondent's health status during the past year. For each item, scores are coded, summed and transformed to a scale from 0 (worst possible health state measured by the questionnaire) to 100 (best possible health state). Scores can be aggregated to measures representing a physical health summary scale (consisting of physical functioning, physical role, pain and general health) and a mental health summary scale (vitality, social functioning, emotional role, and mental health) [6].

The answers to the question of SF-36 about self reported changes in health status, ("compared to one year ago, how you would rate your health in general now") were dichotomized in better, about the same or worse than one year ago.

### Activities of Daily Living (ADL)

Ability to handle personal and instrumental activities of daily living (ADL) was assessed by a questionnaire that evaluates the functional independence of the individual in performing personal activities of daily living (ADLP) and instrumental activities of daily living (ADLI). The ADLP considered were bathing, dressing, going to the toilet, transferring from bed to chair, continence and feeding. The ADLI considered were cleaning, food shopping, public transportation and cooking. This questionnaire was based on Katz' Index of Independence in ADL [22] combined with Hulter Asberg's Instrumental Index of Independence in ADL [30].

Participants were asked whether they were able to perform each task independently. Answers were categorized into two groups, able or unable to perform each activity and group of activities. Patients were classified by their ability to perform physical and psychosocial ADL and four categories were possible: (a) ADLI and ADLP independent, (b) ADLI dependent but ADLP independent, (c) ADLP dependent but ADLI independent and (d) both ADLP and ADLI dependent.

### Employment Status

Patients were asked to classify their employment status as: (a) full-time or part-time; (b) home worker, in lieu of paid employment; (c) unable to work owing to health problems; (d) retired because of age; or (e) unemployed.

### Statistical methods

The baseline demographics of the patients studied were compared with those of all surviving patients available for follow-up. Medians and ranges were calculated for the SF-36.

Descriptive analyses of variables were used to summarize data and the Mann-Whitney U test was used to compare continuous variables between two groups of subjects; chi-square or Fischer's exact test were used to compare proportions between two groups of subjects.

Internal consistency was assessed by Cronbach's alpha in each SF-36 domain.

Three multiple logistic regression analyses with a stepwise forward method, an entry criterion of p < 0.05 and a removal criterion of p < 0.1, were performed with independent variables: SAPS II, age, gender, ASA-PS, type and magnitude of surgery, ICU LOS, marital and employment status; as dependent variables changes in health status question, dependency on ADLP and ADLI tasks were considered.

The odds ratio (OR) and its 95% CI were calculated. Data were analyzed using SPSS for Windows version 13.0 (SPSS, Chicago, IL).

## Results

During the study period, 375 patients were admitted to the ICU. Sixty-four percent were male, the median age was 66 years (minimum 23, maximum 94), median SAPS II was 21 (range 2–82) and median LOS in the ICU was 1 day. Twenty-five (6.7%) patients died in the ICU and a further 17 (11.2%) died on the ward after discharge from the ICU (table 1).

**Table 1:** Characteristics of patients admitted to the ICU (n = 375)

Variable	
Age in years, median (P25–75)	66 (55–74)
Age group, n (%)	
≥ 65 years	201 (54)
< 65 years	174 (46)
Sex, n (%)	
Male	240 (64)
Female	135 (36)
Body Mass Index in Kg/m^2^, median (P25–P75)	24.8 (22–28)
ASA physical status, n (%)	
I	22 (6)
II	138 (37)
III	178 (47)
IV	37 (10)
Type of surgery, n (%)	
Emergent	68 (18)
Scheduled	307 (82)
Magnitude of surgery, n (%)	
Minor	28 (7)
Medium	145 (39)
Major	202 (54)
SAPS II, median (P25–75)	21 (15–31)
Days of ICU length of stay, median (P25–75)	1 (1–3)
Days of ICU length of stay, n (%)	
≤ 7 days	326 (87)
> 7 days	49 (13)
Days of hospital length of stay, median (P25–P75)	17 (9–31)
Mortality in ICU, n (%)	25 (7)
Mortality in hospital, n (%)	42 (11)
Mortality 6 months after ICU discharge, n (%)	38 (10)

ASA, American Society of Anesthesiologists; SAPS II, Simplified Acute Physiology Score, ICU, Intensive Care Unit; P25 and P75 are the 25^th ^and 75^th ^percentiles.

Three hundred thirty-three patients were discharged from hospital; 38 (10.1%) died before the 6-month evaluation (21% of global mortality ratio at the time of evaluation). Of the remaining 295 patients, 69 (23%) did not answer the questionnaire but were known to be alive. The characteristics of all patients still alive at 6-month follow-up are presented in table 2. Participating patients (response rate 77%) had a significantly longer hospital LOS than non-participants and there were no statistically significant differences between participants and non-participating patients regarding age, sex, Body Mass Index (BMI), ASA-PS, ICU LOS or SAPS II.

**Table 2:** Comparison of characteristics between respondents and non-respondents

**Variable**	**Respondents (n = 226)**	**Non-respondents (n = 69)**	**p value**
Age in years, median (P25–75)	65(55–72)	65(52–75)	0.062^b^
Age group, n (%)			
≥ 65 years	112(50)	34(49)	
< 65 years	114(50)	35(51)	
Sex, n (%)			0.183^a^
Male	145 (64)	49 (71)	
Female	81(36)	20 (29)	
Body Mass Index in Kg/m^2^, median (P25–P75)	24.8 (22.6–28.1)	25.7 (23.0–27.8)	0.461^b^
ASA physical status, n (%)			0.894^a^
I	14 (6)	6 (9)	
II	90 (40)	26(38)	
III	107 (47)	33 (48)	
IV	15 (7)	4(6)	
Type of surgery, n (%)			0.459^a^
Emergent	33 (15)	9(13)	
Scheduled	193 (85)	60 (87)	
Magnitude of surgery, n (%)			0.178^a^
Minor	18 (8)	8 (12)	
Medium	96 (42)	21(30)	
Major	112 (50)	40(58)	
SAPS II, median (P25–75)	20 (14 – 27)	18, (12 – 25)	0.606^b^
Days of ICU length of stay, median (P25–75)	1 (0.81–2)	1 (0.88–3)	0.149^b^
Days of ICU length of stay, n (%)			
≤ 7 days	207 (92)	64 (93)	
> 7 days	19 (8)	5 (7)	
Days of hospital length of stay, median (P25–P75)	16 (9–26)	25 (23–28)	0.005^b^

^a ^Pearson χ^2^. ^b ^Mann-Whitney test.

BMI, Body Mass Index; ASA, American Society of Anesthesiologists; SAPS II, Simplified Acute Physiology Score, ICU, Intensive Care Unit; LOS, Length of ICU stay; P25 and P75 are the 25^th ^and 75^th ^percentiles.

Of the 226 participants, 64% were male and the median age was 65 years. Most (54%) were considered ASA-PS III or IV and 85% were submitted to scheduled surgery, 50% to major surgery; the median SAPS II was 20 and only 8% stayed in the ICU for longer than 7 days.

### Employment Status

Twenty-four (24%) patients classified themselves as being in full or part-time employment, 13 (6%) as housekeepers and 153 (68%) as retired.

### Marital status

Sixty-five (30%) patients were single, divorced or widowed and 155 (70%) were married or unmarried couples living together.

### Quality of Life Measures

Overall, 59% stated that their level of health in general was better on the day of testing and 20% considered their level of health in general to be worse at that time than previously (6 months before ICU discharge). There was no statistically significant relationship between the patient's baseline characteristics and a worse self -reported general level of health.

Although all respondents were surgical patients, our hypothesis was that patients could be grouped by characteristics represented by background data variables (gender, age, ASA-PS, BMI, type and magnitude of surgery), data collected during ICU and hospital stay (severity of illness, ICU LOS and hospital LOS) and employment and marital status data collected 6 months after ICU discharge.

Amongst women, there were no statistically significant differences in any domain between the two age groups. In contrast, older men (≥ 65 years), demonstrated significantly better health with respect to the vitality domain than their younger counterparts (table 3).

**Table 3:** Median and sample sizes for the eight domains of the short-form 36 (SF-36) by gender and age (< 65 years and ≥ 65 years)

	**Total**	**Men (n = 145)**			**Women (n = 81)**		
	(n = 226)	Age <65 years (n = 77)	Age ≥ 65 years (n = 68)	p^a^	Age <65 years (n = 37)	Age ≥ 65 years (n = 44)	p^a^
Physical functioning	55	60	55	0.692	55	45	0.308
role physical	44	38	50	0.117	44	34	0.138
Bodily pain	52	62	73	0.450	51	43	0.332
General health perception	45	50	50	0.938	45	38	0.063
Vitality	33	33	42	**0.036**	33	29	0.078
Social functioning	63	63	63	0.069	63	50	0.463
Role emotional	50	50	50	0.532	50	42	0.188
Mental health	48	48	60	0.080	44	46	0.801

^a ^Mann-Whitney test

There were no differences in any SF-36 domain for either age group. Women had significantly lower scores for bodily pain, general health perception, vitality and social functioning than men. There were no differences in any domain between married patients and unmarried couples living together (table 4).

**Table 4:** Median short-form 36 (SF-36) scores for age, gender and marital status

**SF-36 domains**	**Age (years)**			**Gender**			**Marital status**		
			
	<65	≥ 65	p^a^	Women	Man	p^a^	Single	Company	p^a^
**Physical function**	55	50	*0.334*	50	55	*0.540*	50	55	*0.940*
**Role physical**	38	44	*0.708*	44	44	*0.289*	38	44	*0.904*
**Bodily pain**	52	52	*0.868*	51	62	**0.002**	52	52	*0.834*
**General health**	49	40	*0.259*	40	50	**0.026**	45	45	*0.790*
**Vitality**	33	33	*0.551*	29	38	**0.010**	33	33	*0.896*
**Social function**	63	63	*0.377*	50	63	**0.038**	63	63	*0.873*
**Role emotional**	50	50	*0.809*	50	50	*0.477*	50	50	*0.523*
**Mental health**	44	48	*0.266*	44	48	*0.056*	48	48	*0.987*

^a ^Mann-Whitney test

SF-36, Short-Form-36

ASA-PS showed statistically significant differences in almost all domains: patients classified as ASA-PS III and IV gave worse results than those classified ASA-PS I and II in all domains except bodily pain. There were no statistically significant differences in the median scores for any SF-36 domain regarding magnitude and type of surgery (table 5).

**Table 5:** Median short-form 36 (SF-36) scores for ASA physical status type and magnitude of surgery

**SF-36 domains**	**ASA**			**Scheduled surgery**			**Magnitude**		
			
	I/II	III/IV	p^a^	Yes	No	P	Major	Mediun/minor	p^a^
**Physical function**	65	45	**<0.001**	55	55	*0.842*	55	50	*0.626*
**Role physical**	50	25	**<0.001**	44	38	*0.534*	44	38	*0.350*
**Bodily pain**	62	52	*0.118*	52	52	*0.180*	52	52	*0.286*
**General health**	50	40	**0.001**	45	55	*0.533*	45	45	*0.330*
**Vitality**	42	27	**<0.001**	33	29	*0.204*	33	33	*0.408*
**Social function**	63	50	**0.006**	63	63	*0.999*	63	50	*0.121*
**Role emotional**	67	42	**<0.001**	50	50	*0.733*	50	50	*0.313*
**Mental health**	56	44	**0.001**	48	48	*0.837*	48	48	*0.939*

^a ^Mann-Whitney test

SF-36, Short-Form-36; ASA-PS, American Society of Anesthesiologists physical status.

Patients who stayed in the ICU for more than 7 days had worse scores in all SF-36 domains but statistically significant differences were found only in the role-physical, bodily pain and role-emotional domains. There was no statistical difference between higher SAPS II and worse SF-36 scores except for general health perception. Patients who worked and had employment status had better general health perception (table 6).

**Table 6:** Median short-form 36 (SF-36) scores for length of stray (LOS), SAPS II scores and employment status

**SF-36 domains**	**ICU LOS *(days)***			**SAPS II**			**Employment status**		
			
	< 7	≥ 7	p^a^	< 25	≥ 25	p^a^	Work	Don't work	p^a^
**Physical function**	55	50	*0.612*	55	50	*0.596*	55	55	*0.100*
**Role physical**	44	25	**0.020**	44	38	*0.153*	50	38	*0.173*
**Bodily pain**	52	41	**0.034**	52	44	*0.131*	52	52	*0.263*
**General health**	45	40	*0.936*	50	35	**0.019**	50	40	*0.045*
**Vitality**	33	25	*0.169*	33	33	*0.279*	33	33	*0.343*
**Social function**	63	50	*0.107*	62	50	*0.233*	63	63	*0.107*
**Role emotional**	50	25	**0.015**	50	42	*0.203*	63	46	*0.008*
**Mental health**	48	40	*0.289*	48	48	*0.560*	52	48	*0.125*

^a ^Mann-Whitney test

ICU, Intensive Care Unit; LOS, Length of stay; SAPS II, Simplified Acute Physiology Score.

SF-36, Short-form 36

Only the SAPS II value was associated with worse current self reported general level of health (compared to one year ago) after adjustment for age, gender, ASA-PS, type and magnitude of surgery, ICU LOS, SAPS II and marital and employment status (table 7).

**Table 7:** Logistic regression to evaluate the association between self reported health changes (6 months before ICU admission) and the different variables studied (6 months after ICU discharge)

**Variable**	**Simple OR**	**p**	Adjusted* OR (95% CI)	**p**^a^
Age group				
< 65 years	1			
≥ 65 years	0.917	0.792		
Gender				
Male	1			
Female	1.339	0.387		
ASA physical status				
I/II	1			
III/IV	1.137	0.699		
Type of surgery				
Scheduled	1			
Emergent	2.257	**0.049**		
Magnitude of surgery				
Minor	1			
Medium	1.575	0.501		
Major	1.087	0.902		
SAPS II	1.044	**0.002**	1.06 (1.01 – 1.11)	**0.016**
Days of ICU length of stay	1.194	0.201		
Employment Status				
full employed	1			
part-time employed	0.957	0.949		
housekeepers	0.823	0.651		
retired	0.000	0.999		
Marital status				
single, divorced or widowed	1			
married or unmarried couples living together	1.041	0.914		

^a ^logistic regression analysis with stepwise forward method was used with an entry criterion of p <0.05 and a removal criterion of p < 0.1.

ASA, American Society of Anesthesiologists; SAPS II, Simplified Acute Physiology Score; ICU, Intensive Care Unit; OR, odds ratio

*Adjusted to age, gender, ASA, type and magnitude of surgery, ICU LOS, marital and employment status

The internal reliability of questions in each domain ranged from 0.80 to 0.93.

Six months after discharge from the ICU, 60% and 34% of patients, respectively, were dependent in at least one activity in instrumental and personal ADL.

ASA-PS III and IV patients were significantly more dependent in both ADLP and ADLI than ASA-PSI/II patients. Patients in employment were significantly less dependent in ADLP and ADLI. Older patients were more dependent in both groups but the difference was statistically significant only for ADLI. Women were more dependent than men in both groups but the difference was statistically significant only for ADLP. Patients who had been more severely ill at admission to the ICU were also more dependent, but the difference was significant only for ADLP.

There were no significant differences for the other variables in relation to the measured independence in ADLI or ADLP (table 8).

**Table 8:** Percentage of dependency in ADLI and ADLP

	**ADLI **(% of dependency)	p^a^	**ADLP **(% of dependency)	p^a^
**Age, years**		**0.018**		0.055
< 65	53		29	
≥ 65	67		40	
**Gender**		0.227		**0.020**
Female	64		44	
Male	58		29	
**Marital status**		0.396		0.282
Single	59		31	
Company	61		36	
**ASA-PS**		**<0.001**		**<0.001**
I/II	48		22	
III/IV	70		45	
**Type of surgery**		0.435		0.362
Emergency	60		34	
Scheduled	59		34	
**Magnitude of surgery**		0.183		0.472
Major	56		29	
Medium/Minor	64		41	
**ICU LOS**		0.148		0.497
< 7 days	59		34	
≥ 7 days	74		37	
**SAPS II**		0.066		**0.008**
< 25	56		29	
≥ 25	68		47	
**Profissional activity**		**0.001**		**0.046**
Work	40		23	
Don't work	67		38	
**Total**	60		34	

^a ^Pearson χ^2^

ADLI, Instrumental activities of daily living; ADLP personal activities of daily living; ASA-PS, American Society of Anesthesiologists physical status; SAPS II, Simplified Acute Physiology Score, ICU, Intensive Care Unit; LOS, Length of stay.

Older age and higher ASA-PS were significantly associated with greater dependency in ADLI tasks; for ADLP tasks, only ASA-PS showed a significant association, after adjustment for age, gender, ASA-PS, type and magnitude of surgery, ICU LOS, SAPS II and marital status (table 9).

**Table 9:** Logistic regressions to study the association between greater dependency (on ADLI and ADLP tasks) and the different variables

	ADLI				ADLP			
	**Simple OR**	**p**	Adjusted* OR (95% CI)	**p**^a^	**Simple OR**	**p**	Adjusted* OR (95% CI)	**p**^a^

**Age group**								
< 65 years	1		1		1			
≥ 65 years	1.848	**0.026**	2.36 (1.04 – 5.34)	**0.040**	1.636	0.083		
								
**Gender**								
Male	1				1			
Female	1.292	0.372			1.889	**0.029**		
								
**ASA physical status**								
I/II	1		1		1		1	
III/IV	2.602	**0.001**	3.00 (1.31 – 6.87)	**0.009**	2.803	**0.001**	4.58 (1.68 – 12.46)	**0.003**
								
**Type of surgery**								
Scheduled	1				1			
Emergent	1.002	0.956			1.000	1.000		
								
**Magnitude of surgery**								
Minor	1				1			
Medium	1.418	0.224			1.791	0.067		
Major	1.242	0.677			1.234	0.698		
								
**SAPS II**	1.035	**0.012**			1.033	**0.011**		
								
**Days of ICU length of stay**	1.500	0.421			1.284	0.633		
								
**Employment Status**								
full employed	1				1			
part-time employed	5.000	**0.012**			4.821	**0.014**		
housekeepers	4.208	**<0.001**			2.455	**0.047**		
retired	3.333	0.072			2.679	0.164		
								
**Marital status**								
single, divorced or widowed	1				1			
married or living together	1.132	0.681			1.263	0.0.462		

^a ^logistic regression analysis with stepwise forward method was used with an entry criterion of p <0.05 and a removal criterion of p < 0.1.

SAPS II, Simplified Acute Physiology Score; ADLI, Instrumental activities of daily living; ADLP personal activities of daily living; ASA, American Society of Anesthesiologists; OR, odds ratio

*Adjusted to age, gender, ASA, type and magnitude of surgery, ICU LOS, marital and employment status

## Discussion

A consensus emerging in the literature indicates that mortality and morbidity alone may not be adequate for assessing outcome after surgery [31-33]. Outcome involving HRQOL is patient focused and denotes interest in the patient's perspectives in evaluating health status. In the present study, HRQOL was evaluated 6 months after ICU discharge. The time for assessing HRQOL in follow-up patients was chosen to minimize drop-outs and took account of previous studies suggesting that health problems leading to ICU admission after 6 months are due to chronic underlying conditions, or to new and unrelated health problems commonly encountered in an elderly population [34,35].

Among the patients who completed the questionnaire 6 months after discharge from the ICU, 59% reported feeling better than one year before and 20% reported feeling worse. These findings agree with other reports, using different tools, on patients after ICU discharge [36].

In the SF-36 domains, younger patients reported greater physical and mental problems. Perception of HRQOL in younger patients may be due to a willingness to accept functional limitations or to differences in expectations among younger patients, as stated in the study by Eddleston et al. [37]. This could also explain why, in our results, younger men had the worst scores in bodily pain, role-physical, vitality and mental health. Female patients had significantly worse results in bodily pain, general health, vitality and social function. Impairment of HRQOL with age and gender has previously been reported [36-38] but others have shown no influence of age on HRQOL [39,40].

The ICU variables showed that only ASA-PS physical status was able to predict the lower median results in all SF-36 domains except bodily pain; ASA-PS III/IV patients had significantly lower results in all domains. Although ASA-PS was never intended to be a peri-operative risk score, large studies have suggested that a higher ASA-PS score is one of the best predictors of post-operative morbidity [41,42].

Consistent with a previous study by Graf et al. [18], we found that neither age nor type and magnitude of surgery was associated with differences in HRQOL. For severity of disease measured by differences in SAPS II score, statistical significance was found only for the general health domain.

Previous studies have concluded that pre-existing disease has a significant impact on HRQOL [40] and although pre-existing diseases were not included among the variables considered in the present study, the ASA-PS classification indirectly measured this parameter [43]. Ridley et al. showed that HRQOL scores 6 months after ICU are similar to the pre-ICU scores for patients with pre-existing diseases, but lower in patients suffering acute pathologies [40,44]. This could also explain why logistic regression analysis showed that only SAPS II – when adjusted for age, gender, ASA, type and magnitude of surgery, ICU LOS, marital and employment status – was associated with a decline in self reported general health status, 6 months after ICU discharge compared to 12 months previously (6 months before ICU discharge).

There is no generally accepted definition of the term 'long-term intensive care'. Because of the markedly skewed distribution of LOS-ICU, no obvious cut-off exists and time periods of ≥ 7 days up to > 30 days have been used to define prolonged ICU stay[45,46]. For the present study, greater LOS was defined as an ICU stay of more than 7 days. Longer ICU stay had a significant influence only on the role-physical, bodily pain and role-emotional domains of SF-36. Previous studies have suggested that prolonged ICU LOS does not affect HRQOL after ICU [4,47-49].

In a systematic review of quality of life in adult survivors of critical illness, Dowdy et al. [3] refer to six studies in which survivors of elective versus emergency surgical procedures were evaluated [39,50-53]. In three of these studies [39,50,53], quality of life of life was worse, and in two [36,51], emergency surgical patients had a significantly worse quality of life in a minority of domains. In our study there was no significant association between surgical status (emergency versus elective) and overall HRQOL except in the vitality domain. This could be explained by the fact that 85% of the patients underwent elective surgery.

A limitation of this study was the lack of data about employment status before ICU admission. Our data indicate that most patients were retired 6 months after discharge from the ICU, in contrast to the study of Cuthbertson et al. [54], in which fewer than 60% of patients returned to their previous work after discharge from the ICU.

Global results concerning ADL tasks showed that 6 months after discharge from the ICU, 60% of the patients were dependent in at least one activity in ADLI and 34% were dependent in at least one activity in ADLP. In the original studies of Katz [22] and Hulter Asberg [30] the authors' studied dependency on older patients and results showed these patients to be more dependent than the patients that we have studied. By contrast, the patients described in the study of Niskanen et al. [8], comprised patients admitted to a multidisciplinary ICU and were less dependent.

As in other studies, age, was not a determinant of dependence in ADLP, but patients may still need assistance; and age appears to be determinant of ADLI [55].

The results of the study of ADL may have been influenced by co-morbidities and concurrent diseases; indeed, ASA-PS III/IV patients were more dependent in ADL, in both the ADLI and ADLP tasks. The logistic regression model (adjusted for age, gender, ASA-PS, SAPS II, type and magnitude of surgery, ICU LOS, marital and employment status) showed that SAPS II correlated significantly with disability in ADLP and ADLI tasks, alone for ADLP tasks and combined with age for ADLI tasks. Higher SAPS II scores and higher LOS appeared not to predict disabilities in ADL 6 months after ICU discharge, which could reflect the burden imposed by acute alterations.

Among the hospital survivors, no patients were lost to 6 months follow-up for the assessment of survival, but 23% were lost for the assessment of quality of life because they did not respond the questionnaire. Although the overall characteristics of the non-respondents at discharge from the ICU were similar to those of the participants, it must be emphasized that a poor quality of life or a high incidence of psychological disturbance at the time of the follow-up survey could have contributed to the non-response to the questionnaire and may be seen as a limitation of this study [44,54].

Admissions to ICU are not homogeneous, and generalizing findings to all ICU admissions may be misleading since our sample was representative only of a surgical population. The population studied was composed mainly of patients undergoing scheduled surgery, probably already having a reduced quality of life, surgery being performed as an attempt to improve quality of life and survival.

Eighty-five percent of the patients underwent scheduled surgery, and this should be considered when analyzing our data.

Because HRQOL before admission to the ICU appears to be an important determinant of outcome and HRQOL after discharge [56], another limitation of this study was the absence of any evaluation of HRQOL prior to admission and of a sample to act as control group.

## Conclusion

Most patients who survived after surgical ICU showed a subjective positive perception of HRQOL 6 months after discharge from the ICU.

Some variables collected during ICU stay (ICU LOS and SAPS II) and patients' background data (ASA-PS, age, type of surgery) could be seen as determinants of HRQOL.

Determining functional dependency levels could be considered an indirect evaluation of HRQOL. Six months after ICU discharge, a substantial number of the patients were dependent in at last one activity in personal or instrumental ADL. This may be seen as an indicator of prolonged convalescence in this group of patients.

## Competing interests

The author(s) declare that they have no competing interests.

## Authors' contributions

FA participated in conception, design, acquisition of the data, analysis of the data, statistical analysis, critical revision of the manuscript and supervision.

CS participated in analysis of the data, statistical analyses and drafting of the manuscript.

PM and MC participated in conception, design, acquisition of the data, analysis of the data and critical revision of the manuscript.

HB has been involved in drafting the manuscript, analysis of the data and revising it critically for important content.

All authors read and approved the final manuscript.

## Pre-publication history

The pre-publication history for this paper can be accessed here:


